# A case report of reversible dilated cardiomyopathy due to left main coronary artery ostial stenosis: optimal imaging is key

**DOI:** 10.1093/ehjcr/ytae629

**Published:** 2024-11-23

**Authors:** Alexander J Fletcher, Kieran Bannerman, Emma Finlay, Patrick Noonan, Pankaj Gupta, Mark Richard Davidson, Mark Danton

**Affiliations:** Scottish Paediatric Cardiac Service, Royal Hospital for Children, Glasgow, Scotland, UK; School of Cardiovascular and Metabolic Health, University of Glasgow, Scotland, UK; Paediatric Intensive Care Unit, Royal Hospital for Children, Glasgow, Scotland, UK; Scottish Paediatric Cardiac Service, Royal Hospital for Children, Glasgow, Scotland, UK; Scottish Paediatric Cardiac Service, Royal Hospital for Children, Glasgow, Scotland, UK; Scottish Paediatric Cardiac Service, Royal Hospital for Children, Glasgow, Scotland, UK; Scottish Paediatric Cardiac Service, Royal Hospital for Children, Glasgow, Scotland, UK; Paediatric Intensive Care Unit, Royal Hospital for Children, Glasgow, Scotland, UK; Scottish Paediatric Cardiac Service, Royal Hospital for Children, Glasgow, Scotland, UK

**Keywords:** Cardiac angiogram, Extracorporeal membrane oxygenation, Computed tomography, Ostial atresia, Cardiac catheter, Echocardiography, Case report

## Abstract

**Background:**

Congenital coronary artery anomalies are a rare but reversible cause of dilated cardiomyopathy in infants and children. Optimal imaging strategies to efficiently identify and confirm the diagnosis in the setting of extracorporeal membrane oxygenation (ECMO) are crucial to timely surgery.

**Case summary:**

A 2-month-old boy presented with dilated cardiomyopathy and severe left ventricular dysfunction requiring ECMO support. During an unsuccessful ECMO wean, turbulent flow was noted at the origin of the left coronary artery on echocardiography with subsequent computed tomography (CT) angiogram and cardiac angiogram via catheter confirming the very rare diagnosis of left main coronary artery ostial stenosis. He underwent emergency left coronary artery augmentation with excellent outcome.

**Discussion:**

A high index of suspicion for coronary artery anomalies is required for infants presenting with suspected dilated cardiomyopathy. While CT is a potential diagnostic tool for investigating coronary abnormalities in children, image optimization on ECMO is challenging, with further imaging often required. The superior spatial and temporal resolution of cardiac angiography via catheterization allows definitive diagnosis of coronary artery abnormalities in this situation and facilitates timely surgical intervention.

**Learning points**

- A high index of suspicion is required to make the challenging diagnosis of coronary ostial abnormalities, for which echocardiogram sensitivity is low.
- Performing computed tomography or cardiac angiogram on an ECMO circuit is feasible, but consideration of the most appropriate modality and careful planning of optimal image acquisition is crucial.
- While computed tomography angiography is an emerging diagnostic tool, cardiac angiography via catheter has the highest sensitivity and is the recommended imaging modality for diagnosing ostial coronary abnormalities.

## Introduction

Dilated cardiomyopathy is rare, with 1.1 cases per 100 000 person-years, increasing eight-fold in the infant population (<1 year) and carries with it significant mortality and morbidity.^1^ Screening for reversible causes is therefore standard of care; finding and treating a reversible cause potentially preventing further deterioration and maximizing the recovery of functional myocardium.^2^ We present the case of a 2-month-old infant in whom the coronary arteries arose from the normal position, but multimodality imaging was critical in identifying the rare diagnosis of congenital left main coronary artery (LMCA) ostial stenosis in an infant with suspected dilated cardiomyopathy on extracorporeal membrane oxygenation (ECMO) support.

## Summary figure

**Figure:**
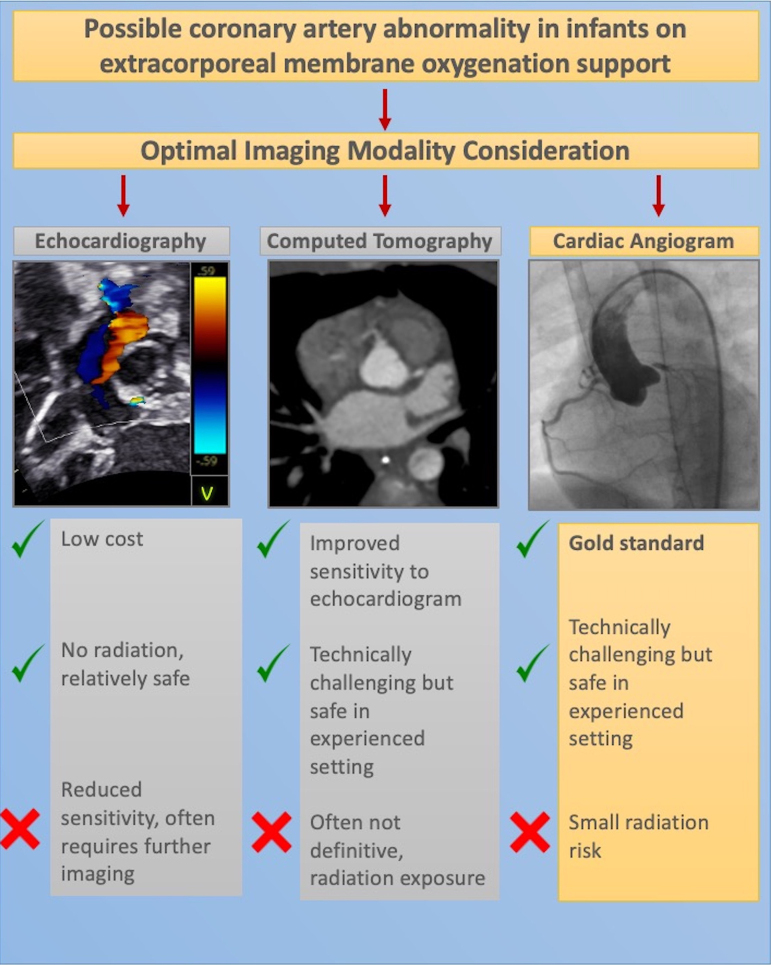


## Case presentation

**Figure 1:**
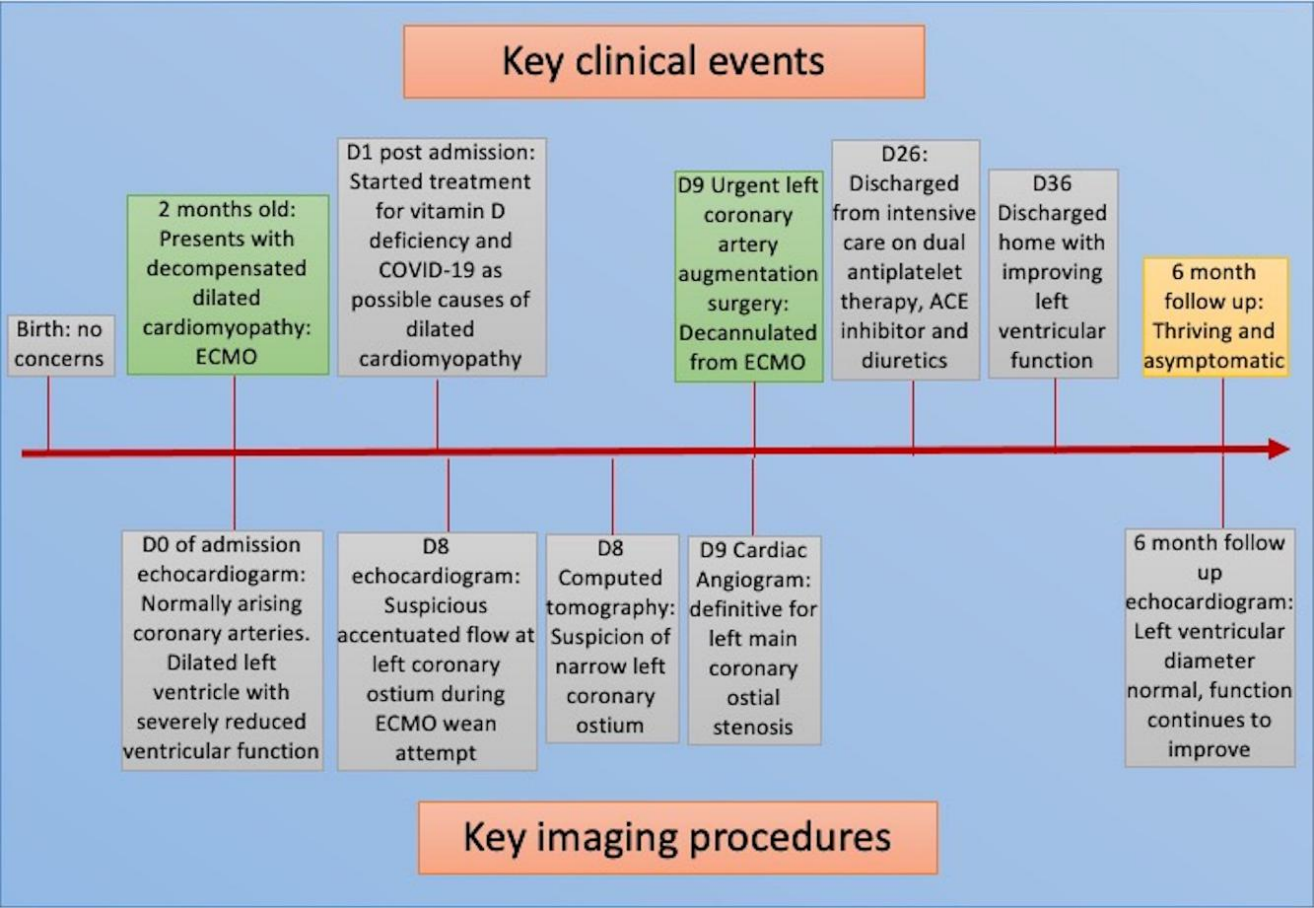
Key clinical events and corresponding imaging results. D0, day of admission; ECMO, extracorporeal membrane oxygenation; ACE, angiotensin-converting enzyme.

**Figure 2:**
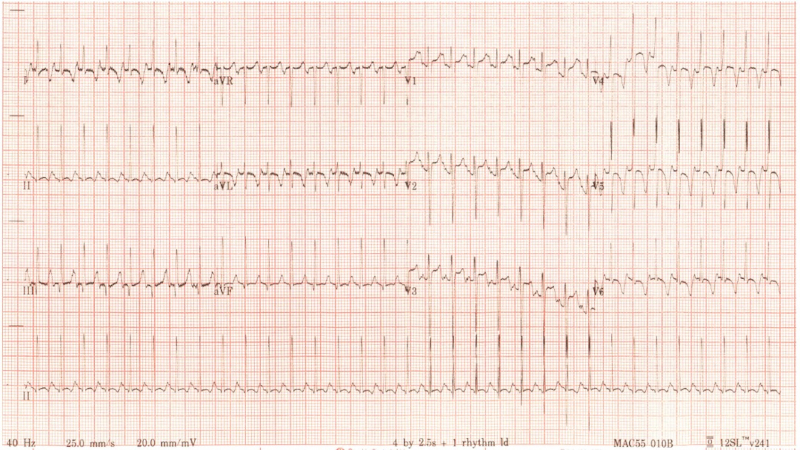
The electrocardiogram at presentation demonstrating sinus tachycardia with lateral T wave inversion, but no deep Q waves in the anterolateral leads suggestive of anomalous left coronary artery arising from the pulmonary artery.

A 2-month-old boy, born at term with no previous medical complaints, was brought by his parents to the paediatric emergency department after 4 days of intermittent tachypnoea and 24 h of reduced feeding (see *Figure 1* for timeline of key clinical events and imaging results). His examination revealed a well grown and non-dysmorphic boy with normal heart sounds; no murmurs and femoral pulses were present but low volume. There was 2 cm hepatomegaly, significantly increased work of breathing with grunting, and bilateral lung crepitations. At presentation, there were signs of shock: tachycardia, hypotension, poor peripheral and central perfusion, elevated lactate, and metabolic acidosis. Electrocardiogram demonstrated lateral T wave inversion, but no clear ST segment changes or Q waves in lead I or AVR (*Figure 2*). A bedside echocardiogram demonstrated dilated left ventricle (end-diastolic diameter 38 mm, *z*-score +8.29), with poor function (global longitudinal strain −1.3%, ejection fraction 28%, fractional shortening 10.5%). Both coronary arteries were seen to arise from the usual sinuses of the aortic root, and the papillary muscles were not echo-bright; therefore, a diagnosis of anomalous left coronary artery from the pulmonary artery was not suspected. Further, there was no coarctation of the aorta; therefore, the suspected diagnosis was dilated cardiomyopathy. The patient’s clinical state warranted dual inotropes of adrenaline and milrinone, as well as emergency intubation and ventilation with the ECMO team on standby. The patient lost cardiac output during the intubation procedure and was emergently cannulated onto VA ECMO (right internal jugular and right common carotid) in the paediatric intensive care unit.

Investigations for dilated cardiomyopathy were performed as part of routine screening including infectious, metabolic, electrolyte, autoimmune, and genetic causes. The high-sensitivity troponin I was 379 ng/L (0–34 ng/L) at admission, fell once on ECMO to 106 ng/L. Other positive results include low levels of COVID-19 detected at point of care testing, treated with 10 days of corticosteroid and two doses of intravenous immunoglobulin, as well as low 25-OH vitamin D levels (16 nmol/L, normal range > 50 nmol/L), treated with 2000 units/day of cholecalciferol. The markers of inflammation were low at presentation, including CRP at presentation of 4 mg/L (0–10 mg/L), normal white cell count of 15.4 × 109/L (6–17 × 10^9^/L), and normal kidney and liver function tests. No other infectious or metabolic causes were suspected through screening making the most likely aetiology for the presentation severe vitamin D deficiency or undiagnosed genetic cardiomyopathy. Serial echocardiograms during the ECMO run demonstrated unchanging dilated left ventricle and consistently poor function (global longitudinal strain −1.3% to −5.2%, estimated ejection fraction 8% to 29%, fractional shortening 2.8% to 11.1%).

**Figure 3:**
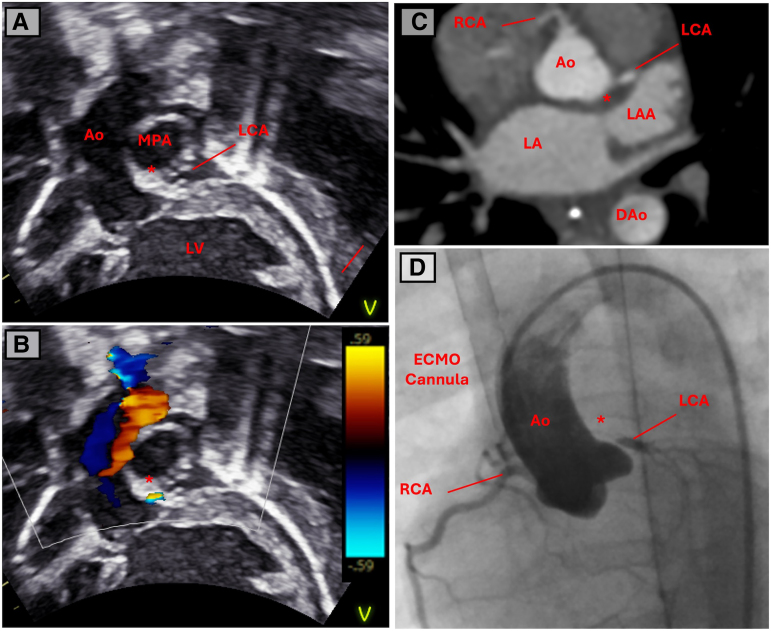
Imaging performed on a 2-month-old with dilated cardiomyopathy on venous-arterial extracorporeal membrane oxygenation support after an unsuccessful decannulation wean. *(A)* 2D subcostal left ventricular outflow view with the distal left coronary artery visible and *(B)* colour Doppler demonstrating turbulent flow between aortic root and visible left coronary artery (asterisk). *(C)* An axial computed tomography image of the aortic root at the level of the coronary artery origin. There is a suspicion of a focal occlusion to flow between the aortic root and left main coronary artery. (*D*) Cardiac angiogram demonstrating a clear stenotic portion of the origin of the left main coronary artery.

During an unsuccessful wean from ECMO flows on Day 7 of admission, repeat transthoracic echocardiography demonstrated accentuated flow in the origin of the left coronary artery (*Figure 3A* and *B*). Computed tomography (CT) with coronary angiography was then performed on ECMO (*Figure 3C*), demonstrating a right dominant circulation and a suspicious of LMCA narrowing, but with satisfactory distal opacification of normal branching left anterior descending and circumflex without obvious collateralization or aneurysms. The most likely diagnosis was congenital left coronary artery ostial stenosis, with ultra-rare entities such as infantile Kawasaki disease or incomplete Kawasaki disease considerations. The patient then underwent cardiac angiography on ECMO (total dose-length product 11 cGy/cm^2^) which definitively demonstrated severe stenosis of the proximal LMCA (*Figure 3D*). The patient was taken to theatre immediately and converted to cardiopulmonary bypass. Following cardioplegic arrest, the aorta was transected above the sinotubular junction and the operative findings confirmed a pinhole stenosis of the normally positioned left coronary ostium as well as severe hypoplasia (almost atresia) of the proximal 3 mm of the LMCA. The LMCA was incised from the aortic sinus to proximal to the bifurcation and augmented with fresh autologous pericardium. The result was excellent, with the patched LMCA easily accepting a 1.5 mm probe, with good flow demonstrated throughout the LMCA, circumflex, and left anterior descending on epicardial echocardiography.

The patient was successfully weaned from bypass in theatre. Post-operatively, he was extubated on Day 5, inotropic support was discontinued by Day 11, and he was discharged to the ward by Day 15. He was discharged home on post-operative Day 27 with recovering left ventricular function (global longitudinal strain −7.9%, left ventricular ejection fraction 44.6%, fractional shortening 17.9%) on routine anti-failure treatment of angiotensin-converting enzyme inhibitor, diuretics, and dual antiplatelet therapy of aspirin and clopidogrel. At 6 months post-operatively, he is thriving without symptoms and his left ventricular function continues to improve with global longitudinal strain recovering to −12.6%, and his left ventricular end-diastolic diameter is now in the normal range of 29 mm (*z*-score +1.72).

## Discussion

Patients presenting with LMCA ostial stenosis/atresia in the first year of life are incredibly rare with less than 30 cases reported worldwide. The presentation is varied, and while majority typically present with heart failure symptoms (62%), a large minority present with cardiac arrest or collapse (31%) while others are identified incidentally in asymptomatic infants (19%).^3–5^ This variety in presentation can present a clinical challenge, and a high index of suspicion is required to make a timely diagnosis.

The diagnostic challenge is compounded by the reduced sensitivity of transthoracic echocardiography in detecting the subtle signs of LMCA ostial stenosis. Of the 21 infants (<1 year of age) with LMCA ostial stenosis/atresia in which the first echocardiogram was reported, the diagnosis was either missed or the coronary arteries reported as normal in 33% of cases.^3–5^ The current case highlights this challenge, with the coronary artery correctly identified to be arising from the normal position on the admission echocardiogram. Once the suspicion of LMCA ostial stenosis is raised in a patient on ECMO, the optimal imaging modality must be carefully considered with CT and cardiac angiography representing two potential adjunctive tools for supporting the diagnosis.

Computed tomography represents a less invasive option that has seen rapidly increasing utilization and is typically the imaging tool of choice when assessing vasculature in infants and children.^6^ While a CT angiogram that delivers a typical slice thickness of 0.6–0.7 mm can identify discrete ostial stenosis in a 1–1.5 mm coronary artery of an infant, interpretation in this population is challenging due to the high heart rate and small calibre vessels (see *Figure 3C*). Therefore, the angiogram needs to be of the highest quality to confidently identify these lesions.^7^ This degree of imaging quality may be technically challenging to obtain in an infant on an ECMO circuit where optimal timing for contrast requires careful consideration (see *Table 1* for a summary of technical considerations). Supporting this, of the 10 cases reported in which CT was utilized for investigating suspected LMCA ostial atresia/stenosis, a further cardiac angiogram was required in eight (80%) cases in order to provide a definitive diagnosis prior to surgery.^3–5^

**Table 1:** Adjunctive imaging in paediatric cardiac patients on ECMO

Modality	Technical considerations	Benefits	Drawbacks
TTE	High index of suspicion required Coronary setting optimization May have limited parasternal views due to ventilation and inability to move patient on ECMO. Consider use of subcostal views including LVOT view to assess coronaries	Cheap Portable with no patient transfer required Relatively safe compared with other imaging modalities No ionizing radiation	Reduced sensitivity and diagnostic certainty Rarely enough to proceed to surgery without adjunctive imaging Operator dependent
CT	Consider injection site^8^: Peripheral line, central line, ECMO cannula directly depending on availability and target vessel to be imaged May need to pause ECMO circuit: up to 100% diagnostic accuracy when 30 s pause used but carries risk of cardiovascular collapse Anatomy in congenital heart disease: abnormal flow can alter ability of contrast to opacify target vessels^9^ Timing trigger: manual, automatic, test bolus or multiple phases. Manual trigger provides control and can prevent early or delayed trigger that can occur with automatic setting Time delays: take account of contrast to vessel of interest as well as trigger to scan start delay Movement of table on acquisition: perform test run before scan. Have perfusionist and physician in the room (lead on)	No extra invasive catheters required Can provide extra information on lung fields, cannula positions Can produce high-quality angiograms when optimal settings used	Requires transport to CT scanner with added risk to patient (although the risk appears low)^10^ Reduced diagnostic certainty in infants due to small vessel size, high heart rate, and ECMO flows. Requires ionizing radiation Pauses in the ECMO circuit
Angiogram via cardiac catheterization	Site of access: new arterial access vs. access through ECMO circuit Pause in ECMO flows: benefits of optimal imaging weighed against risks of pausing Appropriate biplane angulation can be challenging to profile coronary ostia and avoid overlay with ECMO cannulas	Gold standard for diagnosing coronary artery ostial atresia or stenosis High degree of spatial resolution including detailed information on collateral supply Temporal resolution to compare timing of filling of coronaries	Percutaneous access to central artery (e.g. femoral) while anticoagulated on ECMO (risk of vessel injury, bleeding) Access to ECMO circuit entails risk of air entrainment, although this risk is small on the positive pressure side of the circuit Requires transport to catheter suite which carries added risk to patient Requires ionizing radiation

CT, computed tomography; ECMO, extracorporeal membrane oxygenation; LVOT, left ventricular outflow tract

Cardiac angiogram is the diagnostic tool of choice in this condition, confidently demonstrating small or absent connection at the aortic root to the LMCA, as well as delineating collateral supply. It has the benefit of very high spatial and temporal resolution that can help to identify late filling coronary arteries that are supplied by collateralization. In the presented case, the 21 infants described above all received cardiac angiograms, of which 19 were diagnostic (86%) and the other 2 suspicious enough to proceed to surgery. Consideration should, therefore, be given to proceed directly to cardiac catheterization in selected patients and remove the need for CT which may not provide diagnostic certainty. In the current case, the catheter performed on ECMO was diagnostic (*Figure 3D*).

The LMCA ostial stenosis is a rare but potentially reversible cause of dilated cardiomyopathy in infants. A high index of suspicion is required to identify subtle features of the lesion on echocardiography. Once suspected, cardiac angiography via catheterization performed on ECMO is the imaging investigation of choice to facilitate timely surgical intervention.

## Data Availability

Data sharing is not applicable to this article as no data sets were generated or analysed during the current study.

## Lead author biography

Dr Fletcher is a clinical lecturer in Paediatric Cardiology at the University of Glasgow and a paediatric cardiology registrar at the Royal Hospital for Children, Glasgow. He received undergraduate medical training at the University of Dundee, where he completed a BMSc in Neuroscience for which he was awarded the Carl Zeiss Prize. He undertook general paediatric training to registrar level in Glasgow, before completing a PhD in advanced imaging in cardiovascular disease at the University of Edinburgh, for which he has received both national and international awards. Dr Fletcher continues on a clinical academic track, with multiple grants for research focusing on translating novel cardiovascular imaging techniques into clinical practice.

**Consent:** The authors confirm that written consent for the submission and publication of this case, including images, has been obtained from the patient and his parents in line with the Committee on Publication Ethics (COPE) guidelines.

## Notes

**Funding:** A.J.F is supported by the Academy of Medical Science (SGL028\1046 ) and Medical Research Scotland (ECG-1801-2022). Other authors have no declarations.
